# Impact of electrical grounding conditions on plasma–liquid interactions using Thomson scattering on a pulsed argon jet

**DOI:** 10.1038/s41598-021-97185-8

**Published:** 2021-09-07

**Authors:** Elmar Slikboer, James Walsh

**Affiliations:** Centre for Plasma Microbiology, Department of Electrical Engineering and Electronics, The University of Liverpool, Brownlow Hill, Liverpool, L69 3GJ UK

**Keywords:** Physics, Engineering

## Abstract

The interaction between an argon plasma jet excited using microsecond duration voltage pulses and a liquid target was examined using Thomson scattering to quantify the temporal evolution of the electron density and temperature. The electrical resistance between a liquid target and the electrical ground was varied from 1 to $680\, \text {k}\Omega$ to mimic different conductivity liquids while the influence of the varying electrical properties on the electron dynamics within the plasma were examined. It was demonstrated that the interaction between the plasma jet and a liquid target grounded via a high resistance resulted in typical dielectric barrier discharge behaviour, with two discharge events per applied voltage pulse. Under such conditions, the electron density and temperature reached a peak of $1\cdot 10^{15}\, \text {cm}^{-3}$ and 3.4 eV, respectively; with both rapidly decaying over several hundreds of nanoseconds. For liquid targets grounded via a low resistance, the jet behaviour transitioned to a DC-like discharge, with a single breakdown event being observed and sustained throughout the duration of each applied voltage pulse. Under such conditions, electron densities of $2{-}3 \cdot 10^{15}\, \text {cm}^{-3}$ were detected for several microseconds. The results demonstrate that the electron dynamics in a pulsed argon plasma jet are extremely sensitive to the electrical characteristics of the target, which in the case of water, can evolve during exposure to the plasma.

## Introduction

Atmospheric pressure plasma jets are one of the main tools used in the emerging field of plasma medicine and surface functionalization^1–3^. They provide a convenient means to expose a sample to a “cocktail” of radical oxygen and nitrogen species (RONS) under ambient conditions without heating it to extreme temperatures^4^. As many biomedical targets have a significant water content, understanding the interaction between a plasma jet and a liquid target is a precursor to understanding the complex interactions between plasma and living tissues^5,6^.

Underpinning the interaction between plasma and a liquid target are the energetic electrons created within the discharge. It is these that are primarily responsible for the generation of short-lived RONS in the plasma phase which ultimately become long-lived RONS within the aqueous phase and drive the observed biological effects reported in the field of plasma medicine^7^. Critically, the electron kinetics in a non-equilibrium plasma depend on a number of operating parameters (e.g. gas flow, voltage amplitude, frequency, and waveform) and the target characteristics such as the conductivity, grounding arrangement and other material properties^8–12^. A comprehensive discussion on these topics can be found in^13–16^. The influence of the target characteristics on the plasma behaviour is most easily observed through fast imaging of the discharge and gas flow visualization^17^. To understand the electron dynamics more advanced diagnostics are required.

A number of experimental studies have explored the electron dynamics in a kHz-operated plasma jet, these focused either on measuring the peak electric field using techniques as Stark splitting or on the electron density and temperature using Stark broadening and/or Thomson scattering. It has been shown in literature that the behavior of the ionization waves produced by an atmospheric pressure plasma jet differs when a target is present compared to a freely expanding jet^18^. This has a direct consequence for the reactive species’ production, as has been shown for helium metastables when a grounded metal target was placed in front of a plasma jet^19^. Both the plasma plume and the corresponding axial electric field profiles were elongated due to the plasma–surface coupling. Differences between dielectric and metallic targets are known to impact the peak electric field found close to the targeted surface^18^. A liquid target has been shown to elongate the plasma plume and the electric field profile, yet no significant influence of the conductivity of the liquid was found; however, the work employed a liquid target contained within a Petri-dish at floating potential^20^.

Considerable work regarding the electron dynamics, both experimentally and numerically, has been undertaken to characterise short-pulsed ($1\, \upmu \text {s}$, 5 kHz) helium plasma jets expanding freely or interacting with various surfaces^10,21,22^. It has been shown that a return stroke, directly after impact of the ionization waves, occurs when targets are used with a high dielectric constant like water or with a metal.

Indeed, Klarenaar et al. observed that the electron density was reported to rise by a factor of 1.5–2.2 during that return stroke. In those works, the general dynamics of the discharges did not seem to vary dramatically due to the target material properties. For instance, the reported impact of the ionization waves on the target occurred at similar times. After impact, the emission intensity of the plasma plume was observed to rapidly decrease, only with water and a copper targets did some light emission remain a few hundreds of nanoseconds after impact. These observations were attributed to the plasma-target coupling since all targets were at floating potential^10^. An increase of light emission was observed when grounded metal targets were used and therefore at a non-floating potential^19^.

Numerically and experimentally it has been shown that when a metal target is grounded the plasma dynamics change. Viegas et al. highlighted that an increase in current and electron density occur towards the end of the voltage pulse and light emission showed that the plasma was sustained throughout this period. The reported numerical electron densities for that helium jet were $8\cdot 10^{12}$–$8\cdot 10^{13}\, \text {cm}^{-3}$ with an electron temperature ranging between 2.0 and 3.5 eV^22^.

From past works, it is clear that the target characteristics play a crucial role in the resulting dynamics of the plasma–surface interaction. However, an experimental model to examine the various aspects to this phenomenon is difficult to establish; typically, changing the target properties inevitably results in varying a number of parameters simultaneously, e.g. the chemical composition when dealing with a liquid target, secondary-electron emission from a metal target, or the charge deposition on a dielectric target. To simplify the situation, in this work the plasma–liquid interactions were examined between an atmospheric pressure plasma jet and a liquid target (tap water) for which the electrical resistance of the liquid target to ground was varied using an external resistor.

Using this arrangement, changes in liquid conductivity were mimicked without the necessity of altering the liquid composition which could introduce further unknown variables. The adopted configuration reflects many applications in the area of plasma medicine, where moisture-rich targets can be grounded in many different ways ranging from a low impedance direct connection to a floating scenario^23,24^. Understanding how changes to the electrical properties of the target affect the electron dynamics in the plasma is crucial for obtaining a better level of control over the precise exposure the target receives. Short-exposure iCCD imaging and Thomson scattering were used in this work to probe the evolution of the discharge and key discharge parameters, $T_e$ and $n_e$, as the resistance between the target and ground was varied between 1 and 680 k$\Omega$, reflecting both low and high impedance grounding scenarios and the transition between them. The discharge dynamics observed in the 680 k$\Omega$ case was found to be the same as when no ground was added at all to the liquid and therefore the latter was not added distinctively to the manuscript results.

## Results

### Waveforms and light emission

**Figure 1:**
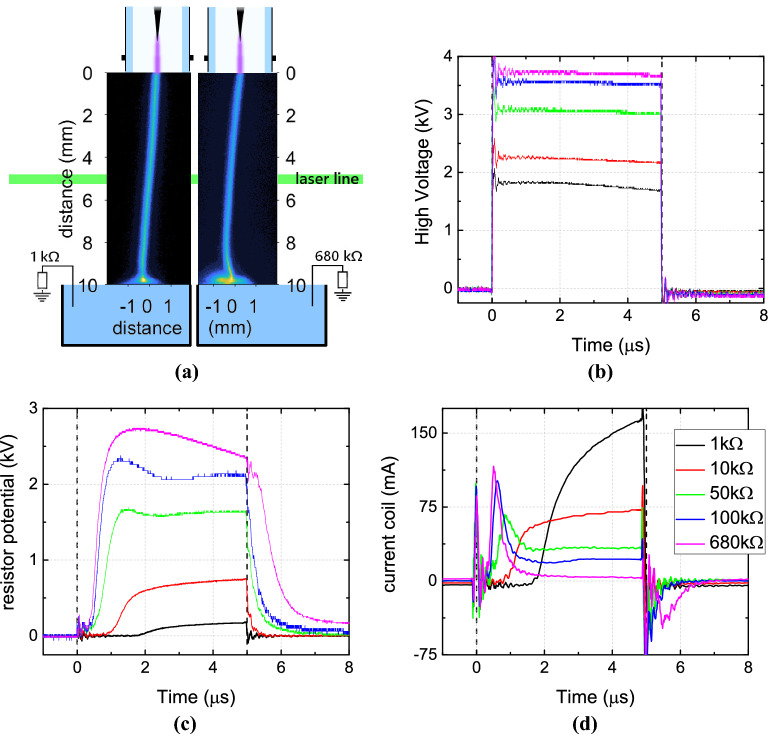
(**a**) two examples of the spatial profile of the light emission from the plasma discharge between the end of the capillary (at 0 mm) and the liquid (at 10 mm) for two cases of $1\, \text {k}\Omega$ and $680\, \text {k}\Omega$ at specific times: respectively at $3.2\, \upmu \text {s}$ and $5.6\, \upmu \text {s}$, using 10 ns exposures. The sketch of the jet and liquid target are present for visualization purposes and therefore out of scale. The waveforms for all five examined cases of varying resistance are shown in (**b**) for the high voltage, (**c**) the potential at the grounded resistor, and (**d**) the current measured with the Rogowski coil.

The plasma jet used throughout this work was a $5\, \upmu \text {s}$ pulsed argon jet operated at 16 kHz. Critically, in order to perform laser diagnostics it was essential for the plasma plume to be spatially and temporally stable, a condition that can only be achieved over a very narrow range of applied voltage settings. It was determined that changes in the grounding resistance of the target necessitated changes in the applied voltage to ensure the stability criteria for laser measurements was maintained. By varying the applied voltage it was possible to operate the jet in a stable mode under all grounding conditions, where the plasma formed a thin ($250\; \upmu \text {m}$) single filament discharge between the capillary of the jet and the liquid. This mode was repetitive in time and space for all cases, see Fig. 1a where 10 ns exposure images are shown of two cases at two different times throughout the applied pulse. Through single shot imaging with a resolution of $25\, \upmu \text {m}$ per pixel, the stability and position of the plasma plume was characterized. In the center of the plume, where the laser scattering was performed, the full-width at half maximum (FWHM) of the light emission was found to be $250\pm 25$ μm. The position of the edges of the FWHM never exceeded variation larger than $25\, \upmu \text {m}$, monitored throughout 30 min of continuous plasma operation. Consequently, under the specific operating conditions of the plasma jet it can be concluded that there is a high degree of spatial and temporal stability, facilitating reliable Thomson scattering measurements. As stated, the behavior of the pulsed argon jet on the liquid surface was examined by grounding the liquid surface using a series of resistors with values ranging from 1 to $680\, \text {k}\Omega$.

The voltage–current waveforms give an initial indication that the dynamics of the plasma were different depending on the resistance used, as shown in Fig. 1. With the liquid target grounded via the lowest resistance, $1\, \text {k}\Omega$, the applied voltage required to obtain a stable discharge was also the lowest at 1.8 kV. Under this condition, the current measured using the Rogowski coil was the highest, reaching a peak of 160 mA reached at the end of the high voltage pulse. This current followed the same temporal profile as that flowing through the grounded resistor from the liquid, shown in Fig. 1c as determined by a potential drop of approximately 160 V. When the resistance at the liquid surface was increased, the plasma dynamics changed and the current detected by the coil differed somewhat from that flowing through the grounding resistor.

Critically, with a high-impedance path to ground, charge delivered to the liquid surface cannot not flow away as easily, this resulted in charge accumulation and an increased potential of the liquid. Due to this phenomena, a higher applied voltage was needed to obtain a stable discharge, e.g. 3.7 kV with the $680\, \text {k}\Omega$ resistor, as shown in Fig. 1b. For all conditions the initial breakdown voltage of the system was approximately 6 kV. Following breakdown, the applied voltage was reduced to obtain a stable discharge that was free from obvious stochastic streamer-like behaviour resulting in the values seen in Fig. 1b. Under high impedance grounding conditions, the current waveform showed a clear positive peak associated with the pulse rising and and a second negative peak associated with the pulse falling edge. Such waveforms are typical of DBD-like behaviour and have been well documented in the literature for pulsed jet impinging on electrically floating targets^25^.

Despite the different applied voltage in each case, which was necessary to maintain a stable single filament discharge between the capillary and the liquid target, the breakdown voltage and the energy per pulse did not vary significantly between the cases. Using the waveforms from Fig. 1, the instantaneous power was calculated from which the energy per pulse was easily obtained through integration. This revealed that the energy per pulse varied between 0.7 and 0.5 mJ for the 1 and $100\, \text {k}\Omega$ cases respectively while it was 0.25 mJ for the highest impedance case of $680\, \text {k}\Omega$.

**Figure 2:**
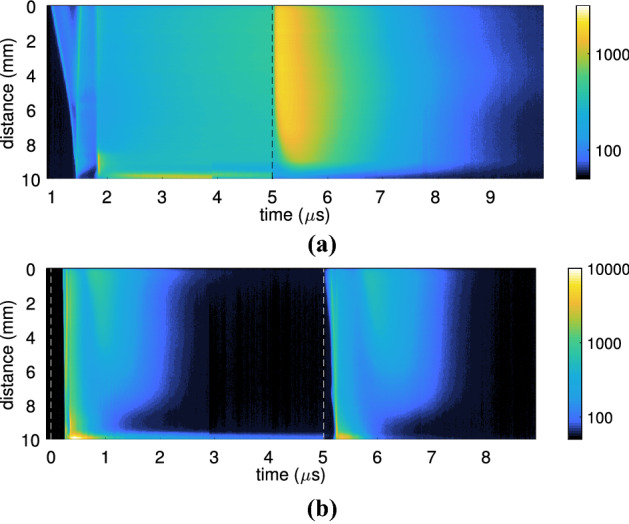
(**a**) The spatio-temporal profile of the light emission from the plasma–liquid interaction when the liquid was electrically grounded through a $1 \; \text {k}\Omega$ resistor. Obtained using fast 10 ns imaging with 480 accumulations each time delay. The end of the capillary was at 0 mm while the liquid was at 10 mm distance. (**b**) The spatio-temporal graph when the resistor was $680 \; \text {k}\Omega$, with the same acquisition settings as (**a**).

A second indication of the induced differences caused by the electrical grounding configuration of the liquid target are seen in Fig. 2a,b. The two figures show the spatio-temporal variation in wavelength-averaged light emission of the plasma jet impinging on the liquid target grounded via a 1 k$\Omega$ and 680 k$\Omega$ resistance, respectively. Using a kinetic series where the time delay was varied in 10 ns steps (equal to the exposure time) images of the plasma impinging on the liquid surface were obtained (480 exposures per frame) and used to create the spatio-temporal emission profile by horizontally binning each image to create a line graph at that specific time delay.

As indicated by the voltage–current waveforms shown in Fig. 1, the plasma dynamics in these two cases were found to be completely different. For the $1\, \text {k}\Omega$ grounding case an ionization wave was seen to propagate from the capillary towards the liquid surface, which it reached at $1.5\, \upmu \text {s}$ during the pulse. Using this information it was estimated the ionization front had a velocity of $2.3\cdot 10^{4}\, \text {m/s}$. Following this, a much faster return stroke was observed leading back to the powered electrode inside the jet capillary, at approximately $1.2\cdot 10^{5}\, \text {m/s}$. At $2\, \upmu \text {s}$ during the applied voltage pulse the main breakdown phase started. This coincided with the sharp rise in current observed in Fig. 1d. It can be seen that the light emission in the plasma plume was almost completely uniform. Only directly at the liquid surface there appeared to be a luminous layer. When the pulse ended at $5\, \upmu \text {s}$ the dynamics changed. The light emissions increased for approximately 300 ns, followed by a gradual decay over a further $2.5\, \upmu \text {s}$.

For the case where a $680\, \text {k}\Omega$ resistor was used to ground the liquid target, a significantly different behaviour was observed. Two clear breakdown events were observed, one during the pulse at $0.5\, \upmu \text {s}$ and one after at $5.5\, \upmu \text {s}$. There was no clear ionization wave travelling from the jet orifice to the liquid surface, as was seen for the lower impedance grounding case. This could have three possible reasons. Firstly, it could indicate that the ionization wave propagated at a velocity in excess of $10^6\, \text {m/s}$. As the spatio-temporal graphs were obtained with a delay step and exposure time of 10 ns, the entire propagation event was captured in a single image frame. Secondly, it could be a result of the jitter in electrical breakdown and thus propagating, which is averaged out due to the number of accumulations (480). Thirdly, there could be sufficient space and surface charge present from previous pulse cycles to prevent a distinct ionisation wave and cause a more homogeneous breakdown along the channel.

Following the breakdown, a secondary afterglow phase appeared to extend from the capillary to within 5 mm of the liquid surface, lasting for approximately $0.7{-}1.0\, \upmu \text {s}$. A similar pattern was observed on the falling edge of the voltage pulse. Consequently, in this grounding configuration no plasma was observed in the discharge gap over a significant portion of the applied pulse (e.g. between 2 and $5\, \upmu \text {s}$). Interestingly, at the liquid surface a luminous layer was observed during the entire applied voltage pulse, although its intensity diminished towards the end of the pulse.

The spatio-temporal emission profiles of the other cases, i.e. with a resistance of 10 k and $100 \; \text {k}\Omega$, are included in the supplementary information(Figures S.1 and S.2). They show a clear evolution from the plasma dynamics observed for the $1 \, \text {k}\Omega$ resistor to the $680\, \text {k}\Omega$ case. When the $100\, \text {k}\Omega$ resistor was used for instance, there were two clear breakdown events similar as with the $680\, \text {k}\Omega$ case (one at $0.5\, \upmu \text {s}$ and another right after the pulse ends at $5.0\, \upmu \text {s}$), but the afterglow was only visible til 2 mm after the end of the capillary. Additionally, towards the end of the applied voltage pulse there was a homogeneous light emission appearing throughout the plasma channel, similar as seen in the 1 and $10\, \text {k}\Omega$ case.

### Evolution of electron density and temperature

**Figure 3:**
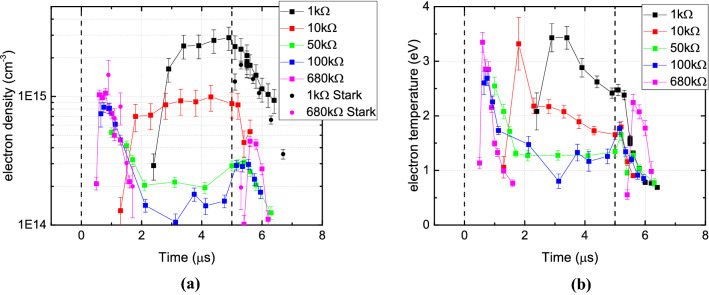
The obtained electron density (**a**) and temperature (**b**) for all five cases of increasing resistance during and after the applied $5\, \upmu \text {s}$ high voltage pulses. For high electron densities (e.g. above $6\cdot 10^{14}\, \text {cm}^{-3}$) 1200 exposures were taken at each data point and 6000 exposures for lower electron densities.

The third and most important indication of the induced differences caused by the electrical configuration of the liquid target are found through Thomson scattering experiments. Figure 3a,b show the temporal evolution of the electron density $n_e$ and temperature $T_e$ in $\text {cm}^{-3}$ and eV, respectively, for the plasma jet interacting with the liquid surface grounded via a resistance varying from 1 to $680\, \text {k}\Omega$. The electron properties were examined in the center of the plasma plume. The temporal behavior of the electron density closely followed the observed dynamics in the current, as shown in Fig. 1d. For the lowest resistance case, the highest electron densities were observed, reaching a peak of $3.0\cdot 10^{15}\, \text {cm}^{-3}$ just before the end of the applied voltage pulse.

With the resistance increased to $10\, \text {k}\Omega$, the peak electron density dropped to $1\cdot 10^{15}\, \text {cm}^{-3}$ but was maintained for a longer duration, spanning over $3\, \upmu \text {s}$. Interestingly, when a $50\, \text {k}\Omega$ resistor or greater was used the temporal profile of the electron density showed a marked difference compared to that observed when a 1 or $10\, \text {k}\Omega$ resistor was used. For cases where the resistance was above $50\, \text {k}\Omega$, the electron density during the main pulse (between 2 and $5\, \upmu \text {s}$) was observed to drop, eventually reaching an undetectable level with the $680\, \text {k}\Omega$ resistor. However, for the higher resistance cases, a current peak appeared shortly after the rising edge of the voltage pulse at approximately $1\, \upmu \text {s}$, during this time peak electron densities of $1\cdot 10^{15}\, \text {cm}^{-3}$ were detected. These densities were not maintained, dropping by an order of magnitude within $1\, \upmu \text {s}$.

To verify the electron density measurements made using Thompson scattering, Stark broadening measurements of the $H_\beta$ spectral line were performed. The electron density determined by Stark broadening for the $1\, \text {k}\Omega$ and $680\, \text {k}\Omega$ is included on Fig. 3a. Under all cases investigated, the intensity of the $H_\beta$ line was low, most likely due to the lack of hydrogen in the feed gas and environment. Consequently, the entire electron density evolution could not be determined using this approach and data points were restricted to time points when the light intensity reached a maximum. Given this restriction, Stark measurements were performed after the $5\, \upmu \text {s}$ for the $1\, \text {k}\Omega$ resistor case and shortly after the voltage pulse rising edge for the $680\, \text {k}\Omega$ case. The electron densities obtained from Stark broadening match closely with the densities obtained from the Thomson scattering experiments.

The electron temperature, shown in Fig. 3b, was found to be the highest at the beginning of the main breakdown phase in all cases. For the larger resistances, the peak occurred at $1\, \upmu \text {s}$, while for $10\, \text {k}\Omega$ and $1\, \text {k}\Omega$ cases, the temperature was seen to peak at $2\, \upmu \text {s}$ and $3\, \upmu \text {s}$, respectively. A peak value of 3 eV was obtained for the higher resistances and a maximum of 3.5 eV for the $1\, \text {k}\Omega$ case. Contrary to the electron density, the electron temperature was not maintained during the pulse for any case. However the decrease of electron temperature depended on the resistance of the target. During the main pulse, an electron temperature of 1.2 eV was observed for the 50 and $100\, \text {k}\Omega$, while 2 and 2.5 eV temperatures were observed for the 10k and $1\, \text {k}\Omega$ case, respectively.

After the pulse ended at $5\, \upmu \text {s}$ it can be seen that only for the higher resistance cases was there an increase of electron density and temperature. For the $1 \,\text {k}\Omega$ and $10\, \text {k}\Omega$ an immediate decay of both density and temperature was observed at the end of the pulse.

## Discussion

In recent years, research focus has somewhat shifted from the investigation of free plasma jets to the more complex case of a plasma jet interacting with a target, a scenario which better reflects the plethora of applications currently being explored. In this work the impact of the electrical grounding properties of a liquid target on the electron dynamics of an argon plasma jet were considered. A pulsed argon plasma jet, excited using $5\, \upmu \text {s}$ voltage pulses at kHz repetition rates was directed towards a liquid target grounded via a variable resistance. The discharge mode was found to depend strongly on the resistance between the liquid target and electrical ground, transitioning from a DBD-like to a DC-like discharge. In all examined cases, the plasma itself appeared as a stable single thin filament that was repetitive in time and space, as shown in Fig. 1a. Additionally, in all cases the non-equilibrium nature of the discharge was maintained, with an approximately comparable energy per pulse being used and the gas temperature monitored to ensure no significant variation.

With the liquid target electrically grounded via a $680\, \text {k}\Omega$ resistor, the discharge dynamics appeared similar to that of a typical DBD, with two distinct discharge events for every single applied voltage pulse. During the applied voltage pulse a current peak was observed when the plasma made contact with the liquid surface; given the high impedance path to ground, this was likely to result in charge displaced by the plasma accumulating at/in the liquid, causing its potential to rise. As in any DBD system, charge accumulation limits the growth of the plasma as it acts to reduce the potential difference across the discharge gap. Under these conditions, light emission from the plasma was confined to the start and end of the applied voltage pulse, while electron densities and energies peaked at $1\cdot 10^{15}\, \text {cm}^{-3}$ and 3 eV, at a point corresponding to the peak discharge current. At the end of the voltage pulse, the applied potential at the powered electrode went to zero and the electrode essentially became a low impedance path to ground. During this time the potential of the liquid had risen to 2.2 kV due to the accumulation of charge, creating a reversal of the electric field and a second discharge to ignite. The electron density and temperature in the second peak was lower than during the first, reaching $5\cdot 10^{14}\, \text {cm}^{-3}$ at a temperature of 2.3 eV.

With a lower resistance between the liquid target and ground, more current could flow and the liquid potential was correspondingly lower. As a result the plasma–surface interaction was different. Following the initial breakdown, the discharge was sustained until the end of the applied voltage pulse indicating that charge accumulation was not sufficiently high to extinguish the discharge.

The electron density grew to $1\cdot 10^{15}\, \text {cm}^{-3}$ and $3.0\cdot 10^{15}\, \text {cm}^{-3}$, respectively for the 10 k and $1 \, \text {k}\Omega$ case, over several microseconds while the current did not pass 170 mA. Under such conditions, the plasma–liquid interaction no longer resulted in DBD-like behaviour, but rather yielded a DC-like discharge where the finite impedance of the plasma and target act to limit the current instead of the accumulation of surface charge. However, the jet electrode is still surrounded by a dielectric glass tube, facilitating the accumulation of surface charge. This charge together with the remaining charges in the gas phase after each discharge event are able to assist the breakdown of the plasma in subsequent pulses.

The impact of the remaining charge is particularly apparent when considering the relatively low operating voltage of just 1.8 kV for the $1\, \text {k}\Omega$ case. Typically, an applied voltage in excess of 6 kV was required to achieve breakdown and initiate plasma formation. The presence of a clear ionization wave (referred to in the past as a *plasma bullet* or *plasma stream*^26^) in the $1 \, \text {k}\Omega$ case compared to the case of higher resistance is likely to be linked to the amount of leftover charges in the system from previous pulses. The dependence on previous discharge events is often referred to as the *memory effect* in the literature, as it contributes to the initialisation of a plasma jet to form stable ionisation waves^27^. Unfortunately, it is difficult to quantify the memory effect as the measurement of such charge between pulses is a very complex undertaking.

Few previous studies have considered the electron dynamics in a plasma jet configuration similar to that considered in this study, consequently there is a limited amount of literature to which a direct comparison of the results can be made. This is common challenge in the field of plasma jet research due to the wide variety of operational parameters that can be used in such a system; for example, a significant amount of work has been done on plasma torches but such discharges are hot and typically use high power radio or microwave frequency sources^28–30^. As a result the reported electron densities and temperatures are not comparable to those observed in this study, i.e. $1\cdot 10^{14}$–$2\cdot 10^{15}\, \text {cm}^{-3}$ and 0.3–1.5 eV. Other jet systems excited using similar kHz frequencies were either examined without a target present^31^, resulting in $2\cdot 10^{12}$–$7\cdot 10^{13}\, \text {cm}^{-3}$ and 0.2–4.5 eV, or using helium instead of argon^32^.

A similar plasma jet system was used in the previous work of the authors; however, it was operated using kHz-sinusoidal excitation and impinged on a liquid target at either a floating potential or directly connected to the electrical grounded^33^. Similar to the observations in this study, when the AC plasma jet interacted with a grounded liquid target a clear plasma bullet was seen to propagate from the capillary to the liquid surface before making contact, with a return stroke following immediately after; such behaviour was not observed when the liquid was at floating potential. The reported peak electron densities and temperatures are similar despite following the kHz-sinusoidal applied voltage^33^.

Other works reported in the literature for pulsed helium plasma jets impinging on targets made from different materials showed similar dynamics to those observed in this study for high impedance targets^10,21,22^. In those works, various targets e.g. glass, water, and metal were used to demonstrate the impact of the targets properties on the plasma–surface interaction. Since most targets were at floating potential, charge accumulation occurred and thus the dynamics of the plasma jet showed similar DBD-like to that in this work for high-impedance liquid targets. When a grounded metallic target is used the dynamics of the jet are known to change, as was shown numerically^22^. Such findings are in close agreement with the DC-like behavior presented in this study for a low-impedance liquid target. Unfortunately, as helium was predominately used in many past works instead of argon and the pulse width and repetition frequencies were different from those used in this study, a quantitative comparison of the results cannot be made; despite this, the measured electron density ranges, i.e. $8\cdot 10^{12}$–$8\cdot 10^{13}\, \text {cm}^{-3}$ and electron temperature range, i.e. 2.0 and 3.5 eV, is in line with the results obtained in this study, shown in 3b.

The results shown in this work relating the dynamics of the discharge mode to the electrical grounding conditions of the liquid target have non-trivial consequences for applications and plasma control. Firstly, as mentioned, in many applications either liquid targets or targets with a significant water content are used in various grounding conditions. But even if the conditions are set beforehand, they might change during the experiment with drastic consequences. When a non-conductive liquid target is used e.g. deionized water that is grounded through a low impedance connection the plasma–liquid interactions will first follow the DBD-like breakdown as we have shown. However as the experiment progresses and the plasma is feeding charged species to the liquid, the conductivity increases and the breakdown mode will shift to the DC-like behavior shown in this work. Secondly, the influence of the pulse width can be discussed as a function of the electrical grounding conditions of the liquid. Following the results from this work it is evident that for low-impedance grounded targets the pulse width could be an interesting tool as a control parameter since the discharge is maintained throughout the length of the pulse. Its role is limited when the impedance is high due to the DBD-like behavior of the discharge dynamics.

In summary, it is vital to understand the behavior of a plasma device under different operating conditions since they are used in such a broad spectrum of diverse applications, ranging from the treatment of polymer films (high resistance) right through to the direct exposure of chronic wounds (low resistance). Critically, for more conductive grounded targets, since the plasma is sustained throughout the applied pulse, an active discharge is present for a longer time with higher electron temperatures than for low impedance targets. This is likely to directly have an impact to the amount of reactive chemical species that are produced and delivered to a target. Understanding how the dynamics of the discharge change in relation to the target characteristics is a first step to gain a better control of the applied and desired plasma–surface interaction.

## Experimental method and theory

**Figure 4:**
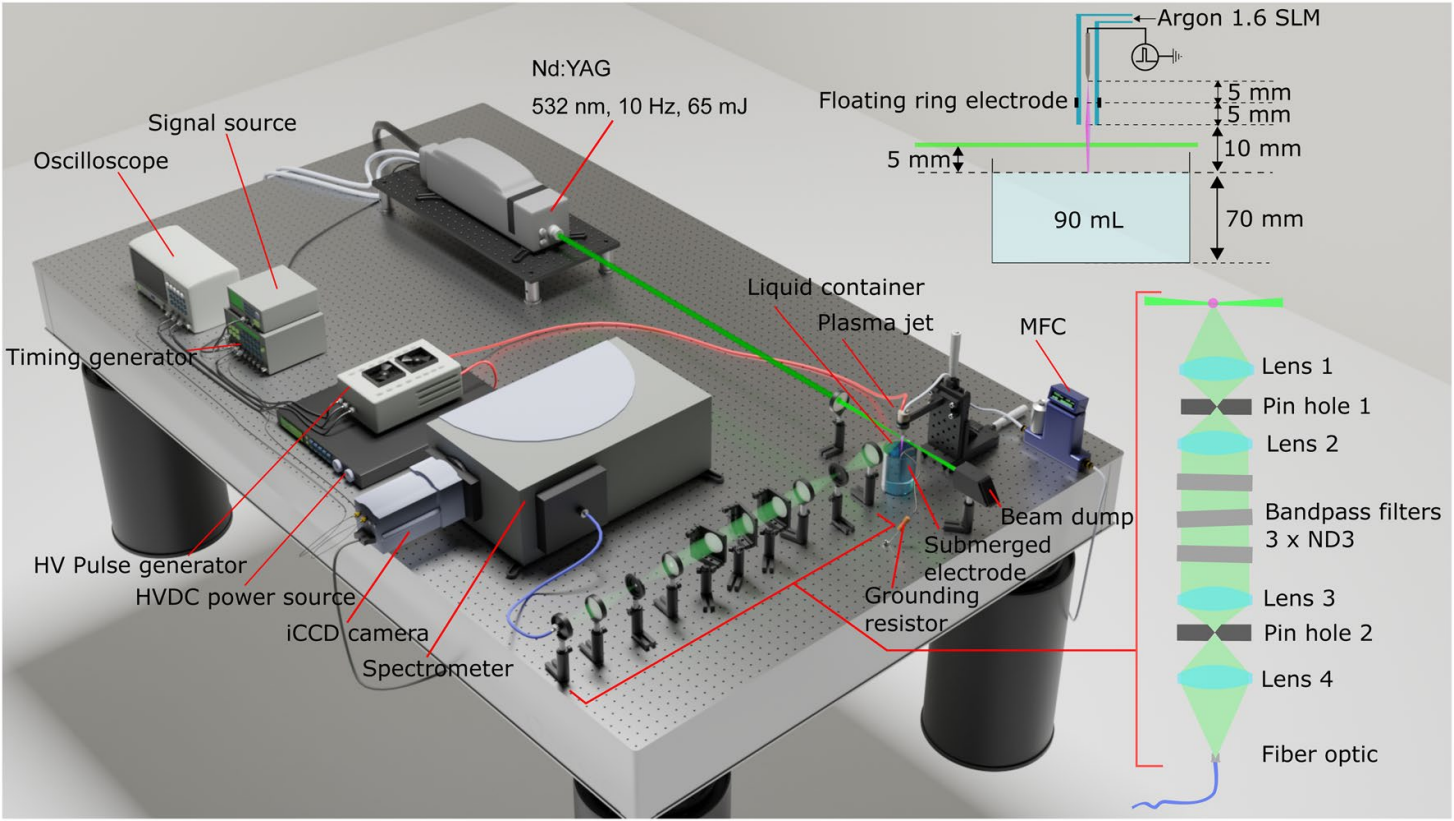
The experimental Thomson setup used throughout this work. The plasma jet is positioned in the focus point of the laser and scattered light is captured at a 90° observation angle. The Rayleigh scatterled light component is filtered out using BragGrate Notch Filters (BNF) as bandpass filters.

The experimental setup used throughout this work to perform the scattering experiments is shown in Fig. 4 and is similar to the setup used previously to investigate a kHz-sinusoidal plasma jet^33^. A 6 ns laser (Litron laser Nano-S-130-10) operated at 532 nm and 10 Hz was focused to a 50 μm spot using a 15 cm lens. The plasma jet system was placed in the focus point of the laser. Three linear translation stages were used to move the jet in all directions with high accuracy. In the horizontal plane of the laser beam, at the focus point, the collecting optics were mounted at a 90° observation angle to capture the scattered light.

The scattered light contains various types of scattering, e.g. Rayleigh, rotational Raman, and Thomson. The Rayleigh scattered light was filtered out by using BragGrate^TM^ Notch Filters (BNF). Three BNFs were used each with an ideal optical density (OD) of 3. The realized filtered OD depends strongly on the angle of the BNF with respect to the collimated scattered light. To achieve a high precision in collimation and remove stray (reflected) laser light, two pinholes were used of 200 μm. A glass fiber directed the scattered light to the spectrometer (Andor SR-500i-A) that was operated using the Andor iCCD camera (DH734-18U-03). The entrance slit of the spectrometer was 300 μm and the grating had 2400 lines per mm.

Both the laser and the Andor camera were operated using an external trigger. A kHz signal was needed to drive the high voltage pulses in the required frequency, while the laser operated at 10 Hz. To achieve appropriate timing of the external trigger signals, several pulse generators were used as shown in Fig. 4. The timing generator (*Stanford DG535*) allowed various signals to be send at a precise and fixed delay from each other. This was necessary to assure that the camera opened the gate at the right time related to the laser pulse and that both could be varied in time with respect to the applied high voltage pulse.

The gating of the iCCD camera was monitored on the Tektronix osciloscope together with the high voltage signal and the current measured using a Rogowski coil. Also the potential difference across the resistor that was attached to the liquid reservoir was captured.

The plasmas jet was mounted vertically 10 mm above the liquid surface (volume glass beaker 90 ml, conductivity tap water $220\, \upmu \text {S/cm}$). Inside the glass capillary of the jet was a stainless steel pin that acted as powered electrode. A metallic ring at floating potential was present at the outside of the capillary tube at 5 mm from the end of the powered electrode, leaving 5 mm till the end of the capillary. Argon gas flowed at 1.5 slm through the capillary in open air at atmospheric pressure. The high voltage amplitude was variable and $5\, \upmu \text {s}$ monopolar positive pulses were applied with a frequency of 16 kHz. The waveforms are shown in Fig. 1. The resistor that was placed between the liquid reservoir and the ground was varied in resistance from 1 to $680\, \text {k}\Omega$ in various steps.

The laser spot and the height of the collecting optics was fixed but the position of the plasma jet and liquid target was variable. In this work the scattered light was examined from a point half-way the plasma plume gap between the exit of the capillary and the liquid surface. Thomson scattering was of the main importance since the electron density and temperature were deduced from it. Rotational Raman scattering of air (without plasma or gas flow turned on) was used for the calibration of the scattering system.

Several theses can be used for the theoretical background on scattering experiments^28,34,35^ hence only a short background will be given here. The intensity detected by the iCCD camera in a scattering experiment is described by

1 $$\begin{aligned} S_\lambda= & {} flI\Delta \Omega \cdot n\cdot \frac{d\sigma }{d\Omega }\cdot G_\lambda (\lambda ) \nonumber \\\equiv & {} C\cdot n\cdot \frac{d\sigma }{d\Omega }\cdot G_\lambda (\lambda ), \end{aligned}$$

with *n* the number density, $d\sigma /d\Omega$ the differential cross-section, and $G_\lambda (\lambda )$ the spectral distribution with normalized area. The calibration constant *C* depends on the efficiency constant *f*, the detection volume length *l*, laser intensity *I*, and detection solid angle $\Delta \Omega$. The type of scattering that is examined determines the differential cross-section and spectral distribution, but the calibration constant C is the same between experiments with consistent acquisition settings. That is why rotational Raman scattering of air can be used as a tool to calibrate *C* used for Thomson scattering to examine the electron density in a plasma.

### Rotational Raman scattering

When a laser beam interacts with air, light is scattered away due to rotational transitions of oxygen and nitrogen molecules that absorb a photon and emit one at a different energy hence wavelength. The resulting Raman shift of wavelength and relative scattered power of each peak (*transition*) can be calculated following the theory in literature^28,34,35^. Therefore the rotational Raman spectrum of air can be modelled and compared with the experimentally obtained spectrum. Since the number density is known the calibration constant *C* is obtained.

**Figure 5:**
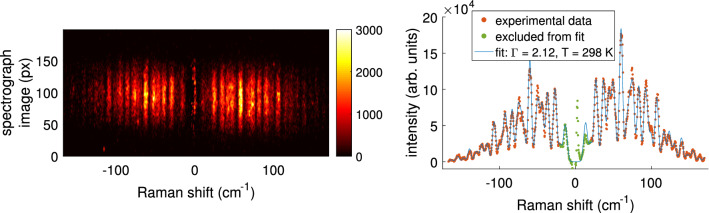
The obtained Raman signal of air, captured using 3000 exposures while the plasma jet and argon flow was inactive. Fitting of the binned spectrum as shown in the right graph allowed to obtain the calibration constant *C* from Eq. (1).

Figure 5 shows the experimentally obtained Raman spectrum of air (left figure), which was vertically binned and plotted in the graph together with the numerically fitted theoretical one. The wavelength was converted to Raman shift in wavenumbers ($\text {cm}^{-1}$). The experimental spectrum was obtained by accumulating over 3000 laser pulses (taking 5 min). The theoretical profile was fitted using Matlab with respect to the Gaussian broadening of the spectral distribution $\sigma =2.12\pm 0.02\, \text {cm}^{-1}$, gas temperature $T=297\pm 4$ K, and signal strength $\text {CA}=(1.85\pm 0.03)\cdot 10^5$ counts. As a result, the calibration constant *C* was known with indication of the uncertainty and could be used from to deduce the electron density from Thomson scattering.

### Thomson scattering

Thomson scattering describes the light scattered by oscillating free charges due to the generated electric field of the laser beam. Following the theory, when a Maxwellian distribution is assumed it can be shown that the Thomson scattered light profile is Gaussian:

2 $$\begin{aligned} G_\lambda (\lambda )=\frac{1}{\Delta \lambda \sqrt{\pi }}\exp \left( -\left( \frac{\lambda -\lambda _i}{\Delta \lambda }\right) ^2\right) \end{aligned}$$

The Gaussian width $\Delta \lambda$ scales directly to the electron temperature $T_e$ according to

3 $$\begin{aligned} T_e=\frac{m_e c^2}{4 k_B}\cdot \left( \frac{ \Delta \lambda }{\lambda _i}\right) ^2 \end{aligned}$$

with $m_e$ the electron mass, *c* the speed of light, $k_B$ the Boltzmann constant and $\lambda _i$ the incident wavelength of the laser. The electron density can be deduced by fitting the Gaussian profile using Eq. (1) to the measured Thomson spectrum since calibration constant *C* is known and the differential cross-section is the electron radius squared.

**Figure 6:**
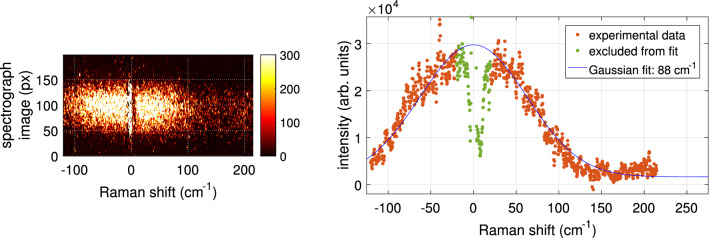
Thomson signal obtained using 3000 exposures taken 800 ns after the start of the applied pulse in the case of 680 k$\Omega$ resistor. The Gaussian fit was used to deduce the electron density and temperature, using the calibration from Fig. 5, resulting in $(1.4\pm 0.1)\cdot 10^{15}\, \text {cm}^{-3}$ and $2.8\pm 0.1$ eV.

An example of an experimentally obtained Thomson spectrum is shown in Fig. 6. The spectrographic image shown on the left was vertically binned and fitted with the Gaussian distribution. Uncertainties are obtained through the fitting procedure and are combined with the uncertainty obtained from fitting the Raman spectrum of air. The resulting electron density corresponds to $(1.4\pm 0.1)\cdot 10^{15}\, \text {cm}^{-3}$ and an electron temperature of $2.8\pm 0.1$ eV. The spectrum was obtained by accumulating over 3000 laser pulses (taking 5 min). For this example the argon plasma jet was interacting with the liquid reservoir being electrically grounded over a 1 k$\Omega$ resistor. The laser and iCCD camera have been triggered to examine the plasma parameters 300 ns before the end of the applied voltage pulse.

### Fast iCCD imaging

In addition to the Thomson measurements to examine the electron density and temperature, also the light emission of the plasma was captured by fast iCCD imaging. The same setup as shown in Fig. 4 was used but the iCCD camera was dismounted from the spectrometer and triggered directly from the same function generator (Tektronix AFG3101C) as used to pulse the plasma. This way the gate of the iCCD camera could be opened at the same frequency (16 kHz) while by varying the internal delay of the camera the entire plasma pulse was scanned. A kinetic series was used for this where the delay is varied in steps of 10 ns (equal to the TTL width). At each delay a frame was obtained by accumulating over 480 pulses. The spatial image of the light emission was summed horizontally at each delay to create a spatio-temporal graph. This was possible because the discharge is a stable thin single filament between the capillary and the liquid which was very repetitive between pulses and experiments.

### Stark broadening

To verify the Thomson measurement results, a second method to determine the electron density has been performed namely based on the Stark broadening of spectral lines of the hydrogen atom^36,37^. The same setup and operation as used for the fast iCCD imaging was used but the iCCD camera was placed back on the spectrometer analyzing the optical emission captured with a optical glass fiber. The spectrometer was operated with a entrance slit of $30\, \upmu \text {m}$ and a grating of 2400 lines per mm. Due to a low optical emission of the $H_\beta$ line at 487 nm a high number of exposures was needed to accumulate enough signal for analysis. This varied between 200 and 480 s of exposure time with the gating of the iCCD camera active for 200 ns each period following the 16 kHz operating signal.

The signal has been analyzed by fitting a Lorentzian profile to obtain a spectral line width. This line width was further analyzed by deconvoluting the van der Waals broadening, the Doppler broadening, and the instrumental broadening. The latter had been estimated to be 0.03 nm by capturing the line profile of a reflected laser beam using the same acquisition settings. The van der Waals broadening and the Doppler broadening depend on the temperature of the heavy particles (i.e. the gas temperature)^36,37^. Since only a simple comparison and verification of the Thomson data is required, the gas temperature is taken at 800 K. This is based on analyses performed on experimental data capturing the optical emission lines of the rotational excited $N_2$ band. At 800 K the van der Waals broadening is equal to 0.038 nm and Doppler broadening to 0.0098 nm. Naturally the temperature of the gas varies in time and depends on the grounding of the liquid. This however is out of the scope of this manuscript. When the temperature is estimated to be too low, it means that the van der Waals broadening is overestimated while the Doppler broadening is underestimated. This error is not important for this work since the Stark broadening results are only used to verify the Thomson results.

## Supplementary Information

Supplementary Information.
