# Supplementary material for: Impact of electrical grounding conditions on plasma–liquid interactions using Thomson scattering on a pulsed argon jet
Source: Sci Rep. 2021 Sep 7;11:17749. doi: 10.1038/s41598-021-97185-8 (PMC8423766; doi:10.1038/s41598-021-97185-8)
Supplement: Supplementary file 1 — Supplementary Information. [file 41598_2021_97185_MOESM1_ESM.pdf]

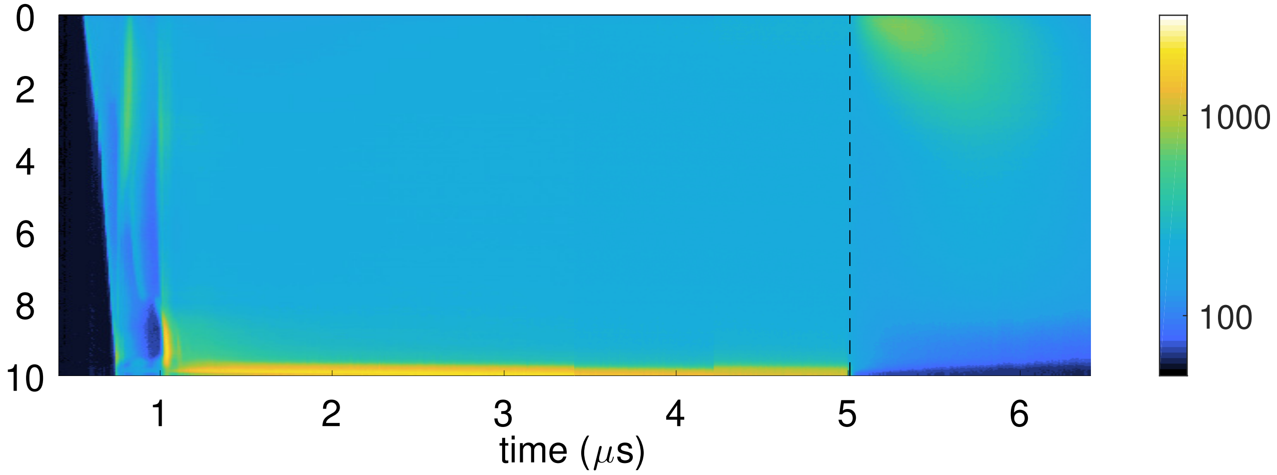

Figure S.1: the spatio-temporal profile of the light emission from the plasma-liquid interaction when the liquid was electrically grounded through a 10kΩ resistor. Obtained using fast 10 ns imaging with 480 accumulations each time delay. The end of the capillary was at 0 mm while the liquid was at 10 mm distance.

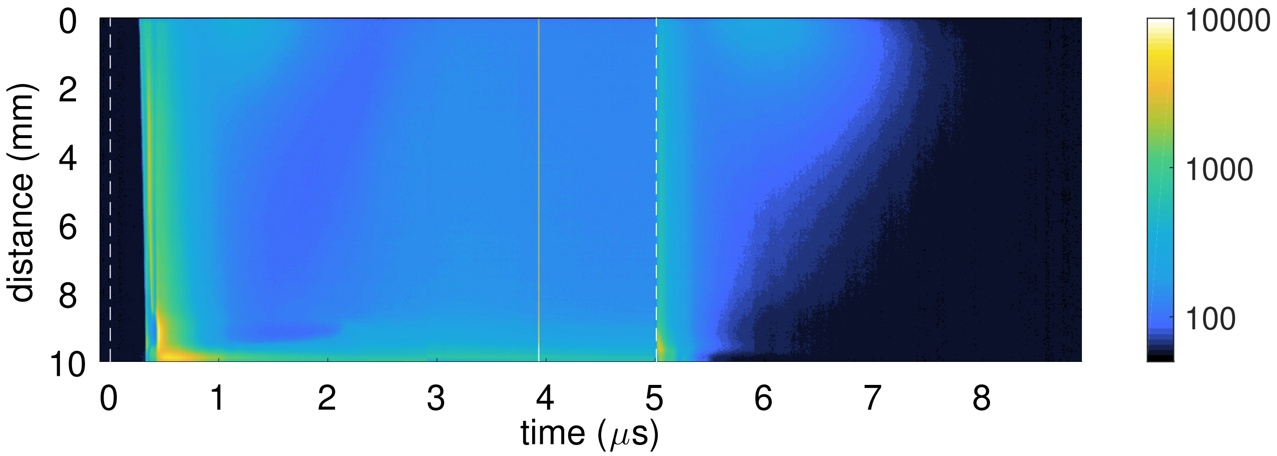

Figure S.2: The spatio-temporal graph when the resistor was 100kΩ, with the same acquisition settings as figure S.1
